# Hypoxia: A Double-Edged Sword During Fungal Pathogenesis?

**DOI:** 10.3389/fmicb.2020.01920

**Published:** 2020-08-12

**Authors:** Hyunjung Chung, Yong-Hwan Lee

**Affiliations:** ^1^Department of Agricultural Biotechnology, Seoul National University, Seoul, South Korea; ^2^Center for Fungal Genetic Resources, Plant Immunity Research Center, and Research Institute of Agriculture and Life Sciences, Seoul National University, Seoul, South Korea

**Keywords:** hypoxia, human fungal pathogens, plant-microbe interaction, plant pathogens, sterol homeostasis

## Abstract

Molecular oxygen functions as an electron acceptor for aerobic respiration and a substrate for key metabolisms and cellular processes. Most eukaryotes develop direct or indirect oxygen sensors and reprogram transcriptional and translational metabolisms to adapt to altered oxygen availability under varying oxygen concentrations. Human fungal pathogens manipulate transcriptional levels of genes related to virulence as well as oxygen-dependent metabolisms such as ergosterol homeostasis when they are confronted with oxygen limitation (hypoxia) during infection. Oxygen states in plant tissues also vary depending on site, species, and external environment, potentially providing hypoxia to plant pathogens during infection. In this review, knowledge on the regulation of oxygen sensing and adaptive mechanisms in eukaryotes and nascent understanding of hypoxic responses in plant pathogens are summarized and discussed.

## Introduction

Oxygen level in natural environments undergoes dynamic changes and provides different oxygen availability to eukaryotes including metazoan, plants, and microbes in their niches. Oxygen availability in a given environment varies greatly from normoxia (basal level of oxygen) to anoxia (naturally oxygen absence). Hypoxia is an intermediate state between normoxia and anoxia, generally described as reduced oxygen availability. It occurs during oxygen depletion, which makes it difficult to perform normal physiological and pathological activities (Ernst and Tielker, 2009; Grahl et al., 2012). Microbes that have diverse habitats, especially pathogenic microbes, are exposed to varying oxygen levels in both terrestrial and host environments having different oxygen states (Ernst and Tielker, 2009; Grahl et al., 2012; Butler, 2013). Hypoxia also occurs at most infection sites and generates significant environmental stress on hosts and pathogens (Mustroph et al., 2010; Burr and Espenshade, 2017). Therefore, out of necessity, eukaryotes evolved sophisticated mechanisms for sensing and adapting to altered oxygen levels. This review gives an overview of the oxygen sensing and signaling mechanisms of eukaryotes and reviews the impact of hypoxia on cellular physiology and virulence in fungal pathogens.

## Oxygen Sensing and Signaling Mechanisms of Eukaryotes in Hypoxia

**Figure 1:**
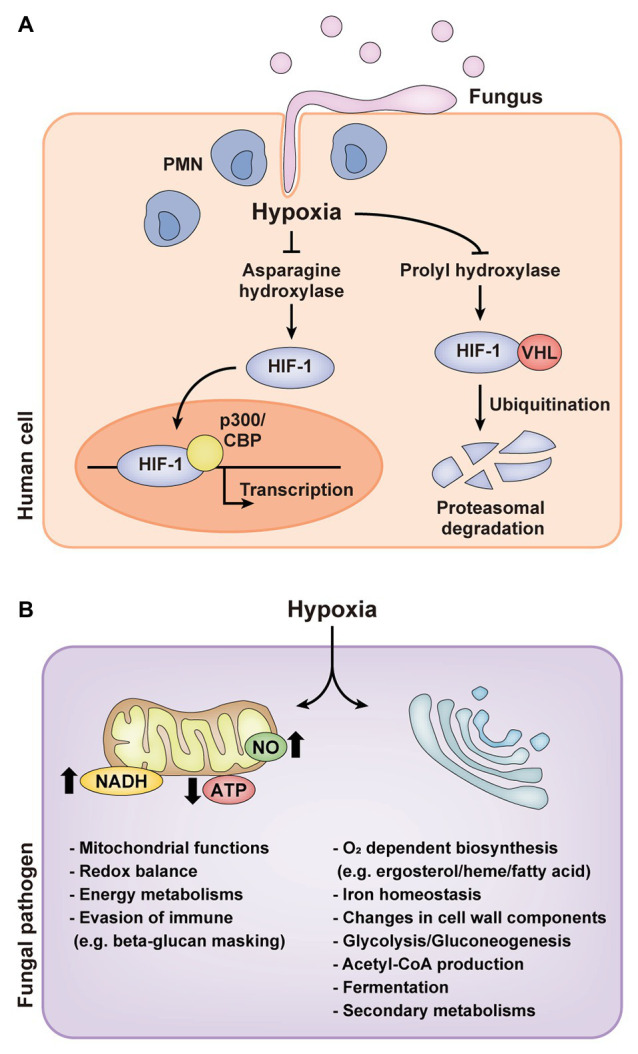
Hypoxic responses of mammals and human fungal pathogens. **(A)** Hypoxia-inducible factor-1 (HIF-1) pathway of mammals in hypoxia during fungal infection. **(B)** Cellular responses of human fungal pathogens in hypoxia. PMN, polymorphonuclear leukocyte; HIF-1, hypoxia-inducible factor-1; VHL, von Hippel-Lindau protein; NO, nitric oxide.

Eukaryotes have acquired multiple oxygen sensors and signal transduction pathways to survive under hypoxic environments. Sensing oxygen changes involves direct sensing by proteins or ligands, directly binding or reacting with oxygen, and indirect sensing by alterations in cellular homeostasis including redox balance and sterol homeostasis (Bailey-Serres and Chang, 2005; Sasidharan and Mustroph, 2011). Mammals and plants, respectively, have direct sensing mechanisms, such as the hypoxia-inducible factor-1 (HIF-1) pathway (Semenza, 1998; Giaccia et al., 2003; Cummins and Taylor, 2005; Bedogni and Powell, 2009; Majmundar et al., 2010), and group VII ethylene response factors (ERFs) regulated by the N-end rule pathway (Gibbs et al., 2011; Licausi et al., 2011; Sasidharan and Mustroph, 2011). In normoxia, HIF-1α is hydroxylated by prolyl hydroxylase domain-containing enzymes (PHDs), which in turn binds with the von Hippel-Lindau protein (VHL) and is degraded in the proteosome by E3 ubiquitin ligase (Bedogni and Powell, 2009; Majmundar et al., 2010). HIF-1α is also hydroxylated by asparaginyl hydroxylase, factor-inhibiting HIF-1α (FIH-1) in normoxia. Hydroxylation of HIF-1α by FIH-1 disrupts the interaction between HIF-1α and co-activators, p300/CBP, inhibiting transcriptional activity of HIF-1α. In hypoxia, proline and asparagine hydroxylation of HIF-1α decreases by inactivation of PHDs and FIH-1, consequently the rate of HIF-1α degradation is reduced and HIF-1α binding with p300/CBP is activated for transcriptional regulation of hypoxia-responsive genes related to redox homeostasis, autophagy, tumorigenesis, and angiogenesis (Figure 1A; Bedogni and Powell, 2009; Majmundar et al., 2010). ERF such as RAP2.12 is continuously expressed and forms a complex with membrane-bound acyl-coA binding proteins (ACBPs) in normoxia (Gibbs et al., 2011; Licausi et al., 2011; Sasidharan and Mustroph, 2011). When exposed to hypoxia, RAP2.12 is dissociated from ACBPs and translocated in the nucleus for activating hypoxia-responsive genes. Upon reoxygenation, the N-terminal methionine of RAP2.12 is cleaved, leading to the oxidation of an exposed cysteine by cysteine oxidases and protein degradation *via* N-end rule pathway (Gibbs et al., 2011; Licausi et al., 2011; Sasidharan and Mustroph, 2011). In contrast, direct sensing mechanisms have not been uncovered in microbes.

Indirect sensing mechanisms have been widely described on mitochondrial reactive oxygen species (ROS) and nitric oxide (NO) productions and sterol homeostasis (Kobayashi et al., 1996; Bailey-Serres and Chang, 2005; Emerling and Chandel, 2005; Castello et al., 2006; Galea and Brown, 2009; Poyton et al., 2009; Takaya, 2009; Osborne, 2011; Waypa et al., 2016; Pucciariello and Perata, 2017). Mitochondria, along with the activation of the plasma membrane NAD(P)H oxidase, have been implicated in activating diverse hypoxic responses through the production of ROS and NO (Bailey-Serres and Chang, 2005; Castello et al., 2006; Waypa et al., 2016; Pucciariello and Perata, 2017). ROS and NO are generated from complex III or IV of the electron transport chain (ETC) and are released to the cytosol during hypoxia. Mitochondrial oxygen-sensing and hypoxic responses are evolutionarily conserved in eukaryotes (Bailey-Serres and Chang, 2005; Castello et al., 2006; Waypa et al., 2016). In mammals, hypoxia-induced ROS, including superoxide anion (O_2_^−^) and hydrogen peroxide (H_2_O_2_), are involved in the stabilization of HIF-1 by inhibiting PHD activity (Majmundar et al., 2010; Waypa et al., 2016). ROS also participate in the activation of other hypoxia-responsive transcription factors, carotid body chemotransduction as an O_2_ sensor, and the release of calcium and other molecules such as cytochrome c (Bailey-Serres and Chang, 2005; Waypa et al., 2016). ROS production in plants under hypoxia is also linked to the activation of transcription factors such as heat shock proteins (Pucciariello and Perata, 2017). This allows plants to acclimate to stress conditions by driving specific developmental processes that improve the transport of oxygen to organs. NO production in hypoxia, intriguingly, contributes to a destabilization of group VII ERFs in the N-end rule pathway through N-terminal cysteine modification as in normoxia (Gibbs et al., 2011; Licausi et al., 2011; Sasidharan and Mustroph, 2011; Pucciariello and Perata, 2017). In *Saccharomyces cerevisiae* and *Fusarium oxysporum* exposed to hypoxia, mitochondrial ROS and NO are also involved in hypoxic signaling (Kobayashi et al., 1996; Takaya, 2009). ROS in *S. cerevisiae*, for example, leads to increased levels of protein and DNA oxidation and increased expression of the oxidative stress-responsive gene, *SOD1* (Dirmeier et al., 2002; Castello et al., 2006). Produced NO combines with superoxide to form peroxynitrite, which promotes protein tyrosine nitration of specific proteins involved in the hypoxic signaling pathway (Castello et al., 2006).

Sterol homeostasis in cells also acts as an oxygen sensing mechanism in eukaryotes. Sterol biosynthesis is a highly oxygen-consumptive process due to oxygen-dependent enzymatic activities. Synthesis of one molecule of sterol requires 11 or 12 molecules of oxygen (Galea and Brown, 2009; Osborne, 2011; Burr and Espenshade, 2017). Sterol regulatory element binding proteins (SREBPs) have been well-studied as principal regulators of sterol biosynthesis in hypoxia and sterol depletion in eukaryotes including fungal pathogens (Emerling and Chandel, 2005; Bien and Espenshade, 2010; Osborne, 2011; Butler, 2013; Burr and Espenshade, 2017). SREBPs are the membrane-bound bHLH transcription factors initially synthesized as inactive precursors and anchored in the endoplasmic reticulum (ER) by SREBP cleavage-activating protein (SCAP) which interacts with ER-resident protein, INSIG. The SREBP-SCAP complex is retained in the ER membrane in normoxia, while SCAP escorts SREBPs from the ER to Golgi where SREBPs are cleaved by protease or Dsc E3 ligase complex for activation in hypoxia. Following proteolytic cleavage activation, activated SREBPs (SREBP N-terminus) are released to the cytoplasm and moved into the nucleus to regulate the expression of hypoxia-responsive genes involved in sterol biosynthesis, lipid and heme biosynthesis, and iron homeostasis. Although SREBP pathway has been conserved in eukaryotes, the components of the SREBP pathway vary among fungi (Bien and Espenshade, 2010; Butler, 2013; Lima et al., 2015). SCAP homologs are conserved, for example, in *Schizosaccharomyces pombe* and *Cryptococcus neoformans*, but absent in *Aspergillus fumigatus* and *Paracoccidioides* spp. (Bien and Espenshade, 2010; Butler, 2013; Lima et al., 2015). The feature of SREBP, a transmembrane helix, is also different among fungi (Chung et al., 2019). SREBPs of *A. fumigatus* (SrbA) and *S. pombe* (Sre1 and Sre2) contain one or two transmembrane helices, but there is no transmembrane helix in *A. fumigatus* SrbB, *C. neoformans* Sre1, and *Magnaporthe oryzae* MoSREs (Chung et al., 2019). In *S. pombe*, SREBP N-terminus is further regulated in the nucleus by prolyl-hydroxylase Ofd1 and its inhibitor Nro1 (Osborne, 2011; Butler, 2013; Burr and Espenshade, 2017). SREBP N-terminus is degraded *via* the proteasome by the oxygen-dependent activation of Ofd1 in normoxia. In hypoxia, however, Nro1 inhibits Ofd1 activation and SREBP N-terminus acts as a transcriptional regulator.

## Hypoxia in Human Fungal Pathogens

Healthy human organs or tissues have diverse oxygen distribution, which ranges from less than 2% to a high of 14% (Table 1; Niehoff and Barnikol, 1999; Ernst and Tielker, 2009; Nizet and Johnson, 2009; Haque et al., 2013; Zeitouni et al., 2016; Komatsu et al., 2017; Friedman et al., 2018). On the other hand, wounds, necrotic tissue sites, tumors, and cancers have an oxygen level of less than 1% (Ernst and Tielker, 2009; Nizet and Johnson, 2009; Chung et al., 2012; Grahl et al., 2012; Haque et al., 2013; Zeitouni et al., 2016; Komatsu et al., 2017; Friedman et al., 2018). Human fungal pathogens located in less oxygenic areas encounter alterations in oxygen availability within human tissues during infection. Oxygen consumption from both the host and pathogen and immune responses of the host also contribute to the rapid decrease of available oxygen at sites of infection by fungal invasion (Grahl et al., 2012; Lopes et al., 2018). Together, these factors potentially lead to hypoxia for fungal pathogens. In invasive pulmonary aspergillosis, for example, hypoxia at the site of infection was detected through a decrease in a bioluminescence signal that requires oxygen for the light-producing luciferase reaction, the hypoxia detection agent pimonidazole hydrochloride (Hypoxyprobe-1), and the fluorescent HypoxiSense probe (Ibrahim-Granet et al., 2010; Grahl et al., 2011). Distinct hypoxic regions during infection were also shown in *Histoplasma capsulatum* via an immunohistopathology and flow cytometry using an *in vivo* hypoxia detection agent (Hypoxyprobe-2; DuBois et al., 2016). Hypoxyprobe-2 was detected within liver granulomas induced by the fungus and the surrounding liver parenchyma, and hypoxia in liver was gradually expanded to the entire liver. Hypoxia within the granuloma was also determined by the increased transcript levels of *HIF-1α*, a mammalian hypoxia-responsive gene, and downstream effector genes (DuBois et al., 2016). In *Candida albicans*, hypoxia measurement using HypoxiSense probe revealed that hypoxia is generated by high infiltration of polymorphonuclear leukocytes (PMNs) to the site of infection in host during fungal infection (Figure 1A; Lopes et al., 2018). These observations suggest that the ability of fungal pathogens to adapt to hypoxia is critical for their survival and pathogenicity. Current understandings of fungal adaptations to hypoxia and the linkage between these mechanisms and fungal virulence are reviewed in the following sections.

**Table 1:** Oxygen concentration in organs and tissues.

Name of organ or tissue	Oxygen concentration (%)	Reference
**Mammals**			
Lung parenchyma; liver; kidneys; heart; circulation	4–14	(Haque et al., 2013)
Brain	0.5–8	(Haque et al., 2013)
Eyes (retina and corpus vitreous)	1–5	(Haque et al., 2013)
Bone marrow	1–6	(Haque et al., 2013)
Adipose tissue	2–8	(Haque et al., 2013)
Skin	6	(Niehoff and Barnikol, 1999; Nizet and Johnson, 2009)
Pancreas	4.2–5.6	(Ernst and Tielker, 2009; Komatsu et al., 2017)
Duodenum	9.8	(Ernst and Tielker, 2009; Friedman et al., 2018)
Intestine	7	(Zeitouni et al., 2016)
**Plants**			
Seed coats	1	(Rolletschek et al., 2002; Geigenberger, 2003)
Silique	6–12	(Geigenberger, 2003)
Phloem	5–7	(van Dongen et al., 2003)
Root meristem	4–10	(Ober and Sharp, 1996)
Tuber (periphery)	8–10	(Geigenberger et al., 2000)
Tuber (center)	2–5	(Geigenberger et al., 2000)

### Aspergillus fumigatus

The airborne mold *A. fumigatus* is a typical saprophytic, obligate aerobe and a facultative anaerobe. *A. fumigatus* could sustain mycelial growth in low oxygen levels, around 0.1–1%, and showed different responses to hypoxia depending on the duration of hypoxic exposure rather than the type of culture condition (Barker et al., 2012; Kroll et al., 2014; Losada et al., 2014; Hillmann et al., 2015). Transcriptome analysis of *A. fumigatus* in hypoxia revealed that hypoxia first affected mitochondrial functions and redox balance (Figure 1B; Losada et al., 2014; Hillmann et al., 2015). Hypoxia negatively affected mitochondrial respiration and the tricarboxylic acid (TCA) cycle, but positively affected the γ-aminobutyrate (GABA) shunt, which bypasses TCA cycle (Barker et al., 2012; Hillmann et al., 2015). Longer hypoxic exposure, in turn, induced transcripts related to oxygen-dependent biosynthesis, including ergosterol and heme biosynthesis, and iron homeostasis and diverse secondary metabolisms such as glycolysis, fatty acid metabolism, and cell wall biosynthesis (Figure 1B; Kroll et al., 2014; Hillmann et al., 2015). These regulatory alterations are closely related to ATP generation and fungal growth in the absence of oxygen. The hypoxic transcription factor SREBP, which regulates ergosterol biosynthesis, has been well-studied in *A. fumigatus* (Willger et al., 2008; Blatzer et al., 2011; Chung et al., 2014). SREBPs in *A. fumigatus*, SrbA and SrbB, are critical for normal fungal growth and disease development in hypoxia by directly regulating genes related to ergosterol and heme biosynthesis, iron uptake, and nitrate assimilation (Chung et al., 2014). SrbB also regulates carbohydrate metabolism independent of SrbA.

Heterogeneity in hypoxia fitness, microbial colony morphologies, and virulence has been reported in isolates of *A. fumigatus*, and significant correlation among hypoxia fitness, alterations in colony morphologies, and virulence was observed (Kowalski et al., 2016, 2019). When comparing *in vitro* hypoxia fitness and *in vivo* virulence of *A. fumigatus* isolates, increased hypoxia fitness was higher virulent in the triamcinolone model of invasive pulmonary aspergillosis (Kowalski et al., 2016). In addition, chronic hypoxia exposure leads to increased virulence of *A. fumigatus*. *A. fumigatus* isolates have two microbial colony morphologies, colony furrowing and percentage vegetative mycelia (white, non-conidiating mycelia, PVM), which are associated with biofilm architecture affected by oxygen (Kowalski et al., 2019). The isolates with high furrowing and high PVM altered inter-hyphal interactions in the developing biofilms, resulting in a loss of vertically polarized growth toward air, increased hypoxia fitness, and consequently enhanced virulence. These results can explain that natural environments varying oxygen tensions can generate genetic and phenotypic alterations of some isolates to thrive within the host and result in the virulence heterogeneity between *A. fumigatus* isolates (Kowalski et al., 2016, 2019).

### Candida albicans

The opportunistic pathogenic yeast *C. albicans* colonizes diverse areas of the human body from aerobic body sites (skin and mucosal surfaces) to multiple internal organs and tissues (gastrointestinal area; Setiadi et al., 2006). In *C. albians*, hypoxia induced the transcription of genes involved in iron metabolism, ergosterol and heme biosynthesis, fatty acid metabolism, and cell wall biosynthesis, but reduced the transcription of genes involved in mitochondrial respiration and the TCA cycle, similar to *A. fumigatus* (Figure 1B; Setiadi et al., 2006; Ernst and Tielker, 2009; Stichternoth and Ernst, 2009; Grahl et al., 2012). Hypoxia adaptation of *C. albicans* is associated with the regulation of hypha formation, which is required for biofilm formation, an important virulence factor (Stichternoth and Ernst, 2009). Hypha formation in hypoxia is regulated by transcription factor Efg1p, which allows hypoxic regulation of about half of hypoxia-responsive genes involved in glycolysis, sulfur and iron metabolisms, and fatty acid biosynthesis (Figure 1B; Setiadi et al., 2006; Stichternoth and Ernst, 2009). Another transcription factor, Ace2p, also regulates hypha formation in response to hypoxia (Mulhern et al., 2006; Ernst and Tielker, 2009; Grahl and Cramer, 2010). Ergosterol homeostasis of *C. albicans* in hypoxia is regulated by Upc2p, a transcriptional regulator of ergosterol biosynthesis, instead of SREBP homologs (Silver et al., 2004; MacPherson et al., 2005; Grahl and Cramer, 2010; Synnott et al., 2010; Grahl et al., 2012). Upc2p is required for growth in hypoxia rather than virulence in *C. albicans*.

Recent studies revealed that hypoxia is generated by high infiltration of PMNs to the site of infection in host during fungal infection (Figure 1A; Lopes et al., 2018; Pradhan et al., 2018). PMNs recognize *β*-glucan on the surface of *C. albicans*, a key pathogen-associated molecular pattern (PAMP), through Dectin-1 on the surfaces of PMNs. However, PMN-induced hypoxia triggers *C. albicans* cell wall masking of *β*-glucan, which in turn hinders PAMP sensing of PMNs (Lopes et al., 2018). Consequently, *C. albicans* can escape the immune surveillance by disturbing specific PMN responses such as phagocytosis, ROS generation, or release of the extracellular DNA traps, resulting in enhanced fungal virulence. Hypoxia-induced *β*-glucan masking is dependent upon mitochondrial and cAMP-protein kinase A (PKA) signaling (Pradhan et al., 2018). Defects in mitochondrial function, hydrogen peroxide production, and cAMP-PKA signaling resulted in inhibition of *β*-glucan masking.

### Cryptococcus neoformans

The encapsulated yeast *C. neoformans* is an obligate aerobe and an opportunistic fungal pathogen. Although optimal growth of *C. neoformans* requires atmospheric oxygen levels (~21%), this fungus needs to have mechanisms to adapt to hypoxia due to its habitats such as the intestinal tract of pigeons and the human lungs and brain (Ingavale et al., 2008; Ernst and Tielker, 2009). Transcriptional profiling of *C. neoformans* in hypoxia revealed that genes involved in hexose uptake, ethanol production, heme and ergosterol biosynthesis, fatty acid metabolism, and iron homeostasis were upregulated, as were the cases for *A. fumigatus* and *C. albicans* (Figure 1B; Chun et al., 2007; Ingavale et al., 2008). In contrast to the hypoxic responses of *A. fumigatus* and *C. albicans*, genes related to mitochondrial function and respiration were upregulated and genes related with cell wall and capsule biosynthesis were downregulated in hypoxia (Chun et al., 2007; Ingavale et al., 2008; Ernst and Tielker, 2009; Grahl and Cramer, 2010; Grahl et al., 2012). SREBP homolog (*SRE1*) in *C. neoformans* is also well known to be responsible for hypoxic growth and fungal virulence by regulating the expression of genes involved in ergosterol biosynthesis and iron uptake pathways (Chang et al., 2007; Chun et al., 2007; Grahl and Cramer, 2010; Grahl et al., 2012). In addition to Sre1p, *C. neoformans* also has a fungus-specific histidine kinase (Tco1p) critical for hypoxic growth and virulence (Chun et al., 2007; Grahl and Cramer, 2010; Grahl et al., 2012). The Tco1p pathway might post-transcriptionally regulate hypoxic adaptation in an unknown manner.

### *Paracoccidioides* spp.

*Paracoccidioides* spp. inhabit in subsurface layers of organic matter in natural environments and cause paracoccidioidomycosis in human lungs which compose low oxygen levels. In both habitats, *Paracoccidioides* spp. must tolerate and overcome hypoxia. Proteomic analysis under hypoxia (1% O_2_) revealed global metabolic changes including proteins associated with energy metabolism, mitochondrial activity, and lipid metabolism (Figure 1B; Lima et al., 2015). In short exposure to hypoxia, proteins involved in energy metabolism, mitochondrial activity, glycolysis, and the TCA cycle were reduced in abundance. In contrast, aldehyde dehydrogenase and long-chain specific acyl-CoA dehydrogenase enzymes were induced, suggesting that the acetyl-CoA is produced *via* acetaldehyde and beta-oxidation pathway rather than glycolysis (Lima et al., 2015). In longer hypoxic exposure, reduced levels of proteins involved in energy metabolism and glycolysis were restored (Lima et al., 2015). However, proteins associated with TCA cycle were still reduced in longer hypoxia; instead, proteins associated with GABA shunt were increased, which is known as an alternative route to the TCA cycle. Enzymes involved in beta-oxidation and in production of ergosterol precursor were also increased. These results suggest that the fungus in hypoxia might be remodeling the fatty acid content of membrane lipids to keep the membrane fluidity (Lima et al., 2015). *Paracoccidioides* spp. have conserved SREBPs, named as *PbsrbA*, similar with other human pathogens, *A. fumigatus* and *C. neoformans* (Chang et al., 2007; Willger et al., 2008; Blatzer et al., 2011; Lima et al., 2015). *PbSrbA* is required for hypoxic adaptation, iron acquisition, ergosterol homeostasis, and tolerance to azole drugs.

### Histoplasma capsulatum

*H. capsulatum*, a causal agent of histoplasmosis, like the other human fungal pathogens, must overcome the hypoxic microenvironments to cause disease (Grahl and Cramer, 2010; Grahl et al., 2012; DuBois et al., 2016). When *H. capsulatum* was exposed to hypoxia, the genes related to ergosterol biosynthesis, the transport of metabolic products, nitrosative stress, and guanine nucleotide exchange were positively regulated under control of *H. capsulatum* SREBP (Srb1) in a time-dependent manner (Figure 1B; DuBois et al., 2016; DuBois and Smulian, 2016). Srb1 is necessary for *H. capsulatum* to quickly adapt to hypoxia, withstand the intracellular environment of macrophages, and successfully cause disease and itraconazole resistance (DuBois and Smulian, 2016).

### Mucor irregularis

*Mucor irregularis*, a causal agent of cutaneous mucormycosis, thrives in a skin where the oxygen pressure is only 41 mmHg (~6% oxygen concentration; Niehoff and Barnikol, 1999; Nizet and Johnson, 2009; Xu et al., 2018). Therefore, *M. irregularis* must overcome hypoxia in the human body to survive. *M. irregularis* showed growth retardation in hypoxia compared to normoxia, suggesting that *M. irregularis* may slowly grow during the invasion of human skin to adapt to the hypoxic skin microenvironment (Xu et al., 2018). According to the transcriptome data, *M. irregularis* may use the intralipid pool and the extracellular lipid absorbed through endocytosis instead of the carbohydrates as energy source during infection (Xu et al., 2018). RNA-seq under 6% O_2_ concentration revealed that genes involved in carbohydrate metabolism such as glycolysis, pentose phosphate pathway, oxidative phosphorylation, and GABA shunt were downregulated, while genes involved in gluconeogenesis, lipid/fatty acid metabolism, beta-oxidation, and endocytosis were upregulated (Figure 1B; Xu et al., 2018). The ergosterol biosynthesis-related genes were not significantly affected by hypoxia, except for genes encoding sterol reductase and a cytochrome P450.

## Hypoxia in Plant Pathogens

Although plants produce oxygen as a by-product of photosynthesis, internal oxygen concentrations can fall to low levels within plant tissues due to a reduced supply of oxygen from the external environment (Geigenberger, 2003; Bailey-Serres and Chang, 2005). These natural environments include flooding, excess rainfall, submergence, waterlogging, soil compaction, and microbial activity. Plants are also faced with oxygen deficiency in different tissue sites and plant species, depending on their physical and physiological characteristics, even under normal oxygenated conditions (Zabalza et al., 2009; Mustroph et al., 2010). Low oxygen levels are found within root meristems, tubers, phloem, and seeds (Table 1; Ober and Sharp, 1996; Geigenberger et al., 2000; Rolletschek et al., 2002; Geigenberger, 2003; van Dongen et al., 2003; Narsai et al., 2011). These internal oxygen decreases occur because of not only high oxygen consumption rates resulting from active cellular metabolism but also poor oxygen diffusion due to the lack of an oxygen circulatory system (Geigenberger, 2003; Bailey-Serres and Chang, 2005; Zabalza et al., 2009; Mustroph et al., 2010). Steep drops in oxygen concentration also occur during the germination of seeds and pollen, tuber growth, and fruit development (Bailey-Serres and Chang, 2005; Mustroph et al., 2010; Narsai et al., 2011). Thus, plant pathogens can encounter alterations in oxygen availability within plant tissues and need to adapt to hypoxia during infection.

## External Hypoxia for Plant Disease Development

**Figure 2:**
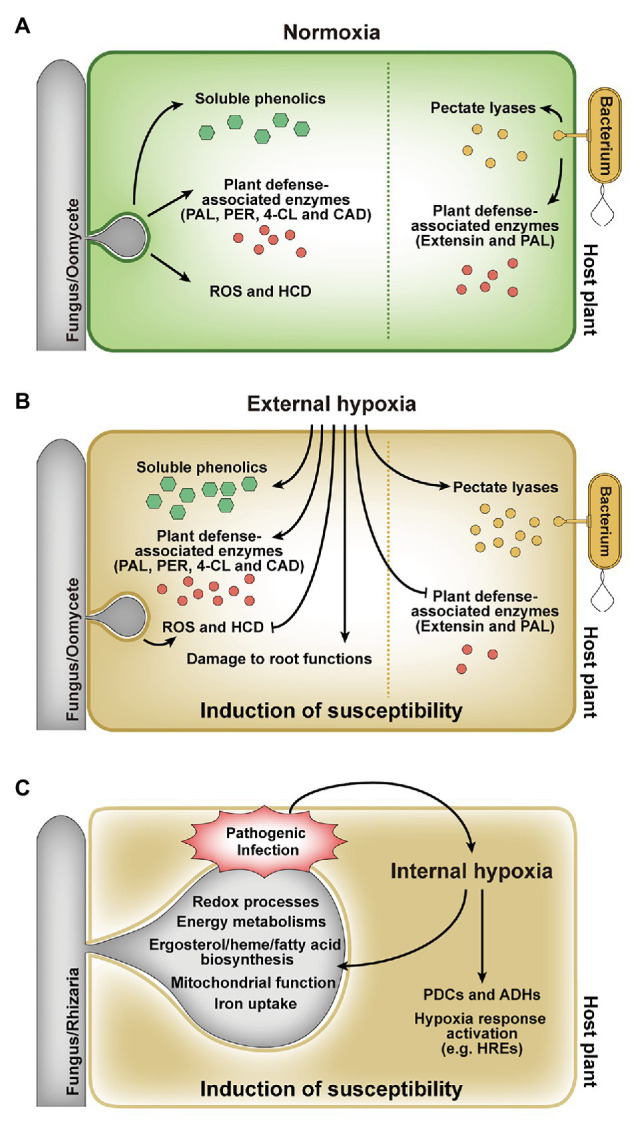
Responses of host plants and pathogens to external and internal hypoxia. **(A)** *In planta* responses during infection under external normoxia. **(B)** Effect of external hypoxia on *in planta* responses during infection. External hypoxia induces host susceptibility by regulating defense responses and affecting tissue functions. **(C)** Effect of internal hypoxia on host plants and pathogens. The pathogen infection leads to internal hypoxia in host plant, and hypoxic environment affects both the host and the pathogen. PAL, phenylalanine ammonia-lyase; PER, peroxidase; 4-CL, 4-coumerate coenzyme A ligase; CAD, cinnamyl alcohol dehydrogenase; ROS, reactive oxygen species; HCD, hypersensitive cell death; PDC, pyruvate decarboxylase; ADH, alcohol dehydrogenase.

The effects of hypoxia on plant diseases have been widely reported in numerous studies over the last few decades (De Boer and Kelman, 1978; Rumeau et al., 1990; Hugouvieux-Cotte-Pattat et al., 1992; Burgess et al., 1999; Aguilar et al., 2000; McDonald et al., 2002; Camy et al., 2003; Babujee et al., 2012; Javid et al., 2013; Moslemi et al., 2018). Hypoxia positively and negatively affects disease development by altering host defense responses (Figures 2A,B). Oxygen status of the tuber, for example, is the critical environmental factor for bacterial soft rot susceptibility and symptom progression upon infection with soft rot *Enterobacteriaceae* (*Pectobacterium* spp. and *Dickeya* spp.; De Boer and Kelman, 1978). Hypoxia altered the activities of pectate lyases secreted by a bacterial pathogen *Erwinia carotovora* and the expression levels of plant defense genes, which resulted in the increased severity of bacterial soft rot on potato tubers caused by *E. carotovora* (Rumeau et al., 1990). Secreted pectate lyases responsible for the maceration of tuber tissues elicited rapid decay of tubers under hypoxia rather than aerobic conditions. Moreover, decreased transcript levels of two plant defense genes, extensin and phenylalanine ammonia-lyase (PAL), in response to hypoxia, also resulted in high susceptibility of tubers to *E. carotovora* infection (Rumeau et al., 1990). Like *E. carotovora*, *Dickeya dadantii* produced increased level of pectate lyase and showed differential expressions of genes associated with anaerobic metabolism under hypoxia (Hugouvieux-Cotte-Pattat et al., 1992; Babujee et al., 2012). Mutagenesis in *Dickeya solani* was also identified hypoxia-related genes involved in fundamental bacterial metabolism, virulence, bacteriocin, and proline transport, which are required for successful disease development (Lisicka et al., 2018). In addition to bacterial soft rot, hypoxia increased the susceptibility of hosts infected with a fungal pathogen *F. oxysporum* and oomycete pathogen *Phytophthora* species (Shivas et al., 1995; Burgess et al., 1999; Aguilar et al., 2000). In banana, cv. Williams is resistant to *Fusarium* wilt disease caused by *F. oxysporum* f. sp. *cubense* (Foc) but succumbs to the disease under hypoxia (Shivas et al., 1995; Aguilar et al., 2000). Although hypoxia stimulated the activities of the plant defense-associated enzymes, PAL and peroxidase (PER), it was not sufficient to prevent disease development. Furthermore, hypoxia might cause irreparable damage to root functions, which may be more susceptible to disease development (Aguilar et al., 2000). *Eucalyptus marginata* showed high colonization by *Phytophthora cinnamomi* and increased susceptibility to the pathogen under root hypoxia caused by waterlogging (Burgess et al., 1999). *E. marginata* in root hypoxia highly activated the plant defense-associated enzymes, PAL, 4-coumerate coenzyme A ligase (4-CL), and cinnamyl alcohol dehydrogenase (CAD), and highly concentrated soluble phenolics (Burgess et al., 1999). However, the relative increase in enzyme activity in response to the pathogen was much lower in plants exposed to hypoxia than normal aerobic conditions, resulting in decreased ability of *E. marginata* to confront the pathogen in hypoxia. This suggests that the enzymes produced during hypoxia might have a more general function than plant defense and are less targeted to the site of infection (Burgess et al., 1999). Similar effects of waterlogging-induced hypoxia on disease severity have been also reported in pyrenthrum infected by the crown and root rot pathogens *F. oxysporum*, *Fusarium avenaceum*, and *Paraphoma vinacea*, and foliar pathogen *Stagonosporopsis tanaceti* (Javid et al., 2013; Moslemi et al., 2018). Waterlogging exacerbates the effect of the pathogens on root expansion, dry weight, and total biomass of root. This synergistic effect may be due to reduction in ATP production by hypoxia, resulting in weakened roots of plant (Moslemi et al., 2018). In addition, hypoxia suppressed oxidative burst and hypersensitive cell death in tobacco and soybean cells inoculated with incompatible isolates of *Phytophthora* species (*P. nicotianae* and *P. sojae*, respectively), which allowed incompatible isolates to infect and colonize host cells (McDonald et al., 2002). Increased disease severity due to hypoxia has also been reported for root rot on apples, cherries, alfalfa, and safflower by *Phytophthora* species (Kuan and Erwin, 1980; Heritage and Duniway, 1985; Wilcox and Mircetich, 1985; Wilcox, 1993; Aguilar et al., 2000). In contrast, hypoxia generated by waterlogging and hydromorphic forest ecosystems has a negative effect on the survival of the root rot fungus, *Collybia fusipes* (Camy et al., 2003). Reduced mycelial growths of *C. fusipes* and another root rot fungus, *Heterobasidion annosum*, under low oxygen condition, supported that the fungal pathogens are highly susceptible to hypoxia (Camy et al., 2003). Hypoxia also negatively affects the biosynthesis of mycotoxins in mycotoxin-producing fungal plant pathogens (Paster and Lisker, 1985; Paster et al., 1986; Tang et al., 2018). The postharvest pathogen *Penicillium griseofulvum* produced less patulin toxin in low oxygen conditions (1 or 5% O_2_ concentration; Paster and Lisker, 1985) and *Fusarium sporotrichioides*, one of *Fusarium* species, produced only trace amounts of T-2 toxin under 5% O_2_ concentration (Paster et al., 1986). *Fusarium graminearum*, a main deoxynivalenol (DON) producer and a causal agent of *Fusarium* head blight in cereal crops, showed less DON production and strongly attenuated virulence under hypoxia but normal radial growth similar to that under normoxia (Tang et al., 2018). The effect of external hypoxia on plant disease development has been studied in numerous plant-pathogen interactions (De Boer and Kelman, 1978; Kuan and Erwin, 1980; Heritage and Duniway, 1985; Wilcox and Mircetich, 1985; Davison and Tay, 1987; Rumeau et al., 1990; Wilcox, 1993; Atwell and Heritage, 1994; Shivas et al., 1995; Burgess et al., 1999; Aguilar et al., 2000; McDonald et al., 2002; Camy et al., 2003; Babujee et al., 2012; Javid et al., 2013; Moslemi et al., 2018), but direct evidence for the occurrence of hypoxia within the plant during pathogenic infection and the effects of hypoxia on both plant host and pathogen have received little attention.

## Internal Hypoxia During Plant-Pathogen Interaction

Pathogen-derived *in planta* hypoxia and the hypoxic responses of both hosts and pathogens have been studied (Hückelhoven et al., 1999; Proels et al., 2011; Gravot et al., 2016; Chung et al., 2019; Valeri et al., 2020). When the parasitic fungus *Blumeria graminis* f. sp. *hordei* infects barley, photosynthetic rates at the site of attack are progressively reduced, hence less oxygen formation (Scholes et al., 1994; Swarbrick et al., 2006; Bischof et al., 2011). Oxygen pressure at the site of attack is further reduced by ROS production at the early stages of infection (Hückelhoven et al., 1999). *B. graminis* f. sp. *hordei* infection also resulted in induced transcript levels of the barley alcohol dehydrogenase (ADH) genes, *HvADH1* and *HvADH2* (Proels et al., 2011). It suggests that the fermentation pathway can be important to maintain the energy metabolism of host under hypoxia during infection. It was also demonstrated that the hypoxia responses of *Arabidopsis* roots are promoted by infection with the gall-inducing protist pathogen, *Plasmodiophora brassicae*, which aids in the development of clubroot, a root gall disease (Gravot et al., 2016). Hypoxia-related genes encoding pyruvate decarboxylase (*PDC1* and *PDC2*) and *ADH1* in *Arabidopsis* were upregulated during *P. brassicae* infection (Figure 2C; Gravot et al., 2016). *PDC1* and *PDC2* mutants showed significantly weak clubroot symptoms. In addition, defects in components of the N-end rule pathway (constitutive activation of hypoxia responses) resulted in enhanced root gall symptoms, while defects in group VII ERF transcription factors (constitutive repression of hypoxia responses) resulted in reduced disease development (Gravot et al., 2016). These results suggest that the hypoxia responses of *Arabidopsis* during *P. brassicae* infection may initially result from competition for oxygen between the host and the pathogen, but consequently results in disease development.

Using non-invasive planar optode technology and geostatistical spatial analysis, it has been recently investigated that behavior patterns of *F. oxysporum* f. sp. *lycopersici* depending on presence and absence of tomato root by visualizing and quantifying the dynamics of O_2_ consumption (Rodeghiero et al., 2018). The air saturation of the pathogen decreased first by the increase in fungal respiration, and then the air saturation of the inner core (root) peaked and decreased during root-pathogen interaction. Afterwards, the air saturation of the pathogen recovered again. *F. oxysporum* f. sp. *lycopersici* tended to grow toward tomato root, in contrast, the fungus moved with a Brownian motion (random) when the root was absent (Rodeghiero et al., 2018). More direct evidences of hypoxia emergence within hosts during fungal infection have been reported in the hemibiotrophic fungus *M. oryzae* causing rice blast (Chung et al., 2019) and the necrotrophic fungus *Botrytis cinerea* causing gray mold (Valeri et al., 2020). In *M. oryzae*, internal oxygen flux in rice tissues infected by the fungus and occurrence of *in planta* hypoxia during fungal infection were observed via two visualizing methods (Chung et al., 2019). A non-invasive optical oxygen sensor indicated that the oxygen concentration within rice leaf tissues decreased by 13–14% during infection with *M. oryzae*, while healthy rice leaves had a 19% oxygen level (Chung et al., 2019). Furthermore, an immunofluorescence assay based on Hypoxyprobe-1 showed that intracellular hypoxia is persisted after fungal penetration (Chung et al., 2019). The occurrence of hypoxia in infected rice was also supported by induced expression of rice genes related to hypoxia during *M. oryzae* infection. These observations directly revealed, for the first time in plants and plant pathogens, that both fungal pathogen and host are able to experience hypoxia during pathogenesis. However, the exact concentration of oxygen in rice cells during fungal infection is still unclear but remains an exciting area for further investigation. Transcriptional profiling of *M. oryzae* in a restricted oxygen condition resulted in the identification of hypoxia-responsive genes involved in mitochondrial function, redox processes, energy metabolisms, ergosterol and heme biosynthesis, fatty acid biosynthesis, and iron uptake (Figure 2C; Choi et al., 2015). These hypoxia responses of *M. oryzae* are conserved in eukaryotes, including human fungal pathogens. Among them, defects in genes encoding SREBP transcription factors (*MoSRE1* and *MoSRE3*) showed attenuated growth under hypoxia, and Δ*Mosre1* displayed reduced invasive growth, suggesting that the SREBP pathway in *M. oryzae* also plays important roles in mycelial growth under hypoxia as well as fungal pathogenicity (Choi et al., 2015; Chung et al., 2019). In *B. cinerea*, local hypoxia in *Arabidopsis* leaves during infection was demonstrated through the expression of hypoxia-responsive genes, GUS reporter system using the promoter of plant cysteine oxidase (PCO), and direct measurement of O_2_ content with an oxygen microsensor (Valeri et al., 2020). There was the induction in the expression of several hypoxia-responsive genes including hypoxia-responsive ERF gene (HRE2) in the leaves infected with *B. cinerea*. O_2_ content at the site of infection was decreased from 18% in control leaves to less than 2% when it was measured with the oxygen sensor (Valeri et al., 2020). Local hypoxia was also confirmed via pPCO1:GUS reporter system based on that PCO1 is strongly and specifically induced by hypoxia (Weits et al., 2014; Valeri et al., 2020). These results suggest that the metabolic activity of the living fungus is responsible for the increase in O_2_ consumption within host and induces local hypoxia at the site of infection in *Arabidopsis* leaves, which is part of defense response of host (Valeri et al., 2020). We have begun to understand the occurrence and effects of hypoxia on plant-pathogen interactions, many questions remain to be answered regarding the adaptive mechanisms of plant pathogens to *in planta* hypoxia and the roles of hypoxia-responsive genes during infection.

## Conclusions and Future Perspectives

In 2019, Nobel Prize in Medicine and Physiology has highlighted the important discovery of oxygen sensing mechanisms and adaptation of eukaryotes to hypoxia, which pave the way for new treatment strategies for anemia and cancer. This review summarized the current knowledge of oxygen sensing and adaptation mechanisms of eukaryotes including fungal pathogens in response to hypoxia. Hypoxia is a common pathophysiological phenomenon with rapid and profound consequences on cellular metabolisms when pathogens infect hosts. Thus, both hosts and pathogens need to adapt to hypoxia by sensing the alteration in microenvironements and manipulating transcripts and proteins related to hypoxia responses. As recent observations in rice-*M. oryzae* and *Arabidopsis*-*B. cinerea* revealed that hypoxia occurs within plant cells during fungal infection (Chung et al., 2019; Valeri et al., 2020), it is imperative to investigate further oxygen-sensing mechanisms and hypoxic adaptations in plant pathogens. However, our understanding of these mechanisms has been limited in human diseases. Mammals and plants have direct sensing mechanisms such as HIF-1 and group VII ERFs, to cope with hypoxia (Bailey-Serres and Chang, 2005; Sasidharan and Mustroph, 2011); however, the direct oxygen-sensing mechanisms of microbes are still undefined and remain to be determined. Hypoxia also rapidly affects oxygen-dependent metabolisms, including mitochondrial functions, heme biosynthesis, and ergosterol homeostasis, which have been reported as an indirect oxygen-sensing mechanisms (Bailey-Serres and Chang, 2005; Emerling and Chandel, 2005; Castello et al., 2006; Galea and Brown, 2009; Poyton et al., 2009; Takaya, 2009; Osborne, 2011; Waypa et al., 2016; Pucciariello and Perata, 2017). Mitochondrial functions such as mitochondria-derived ROS and NO signaling have been well studied as hypoxic signaling pathways and oxidative stress response in *S. cerevisiae* and *F. oxysporum* (Kobayashi et al., 1996; Dirmeier et al., 2002; Castello et al., 2006; Takaya, 2009). However, further detailed studies on mitochondrial functions are still needed in fungal pathogens. Ergosterol homeostasis has also been widely studied as an oxygen-sensing mechanism in fungal pathogens, especially *A. fumigatus*, *C. neoformans*, *Paracoccidioides* spp., and *H. capsulatum* (Ernst and Tielker, 2009; Grahl and Cramer, 2010; Hillmann et al., 2015; Lima et al., 2015; DuBois et al., 2016; DuBois and Smulian, 2016). Once ergosterol homeostasis is altered by hypoxia, SREBP is activated, which regulates ergosterol biosynthesis-related genes for adaptation to hypoxia. The SREBP pathway in those human fungal pathogens is an important determinant of hypoxic adaptation and virulence (Chang et al., 2007; Willger et al., 2008; Ernst and Tielker, 2009; Grahl and Cramer, 2010; Chung et al., 2014; Hillmann et al., 2015; Lima et al., 2015; DuBois and Smulian, 2016). SREBPs in *M. oryzae* are also important transcriptional regulators in the response to hypoxia and are required for growth under hypoxia and pathogenicity (Choi et al., 2015; Chung et al., 2019). Nevertheless, much remains to be learned about the regulatory mechanisms of the SREBP pathway and ergosterol homeostasis under hypoxia in *M. oryzae*. In addition, it is necessary to study other oxygen sensing mechanisms in plant pathogens. Transcriptional profiling under hypoxia revealed that *M. oryzae* also has hypoxia-responsive metabolisms, such as fatty acid and heme biosynthesis, and iron uptake and other transcription factors that are conserved in other fungal pathogens (Choi et al., 2015). It will be intriguing to elucidate their roles in hypoxia adaptation and fungal virulence. Finally, further research on the adaptation mechanisms for *in planta* hypoxia in other pathosystems remains to be investigated.

## Author Contributions

All authors listed have made a substantial, direct and intellectual contribution to the work, and approved it for publication.

### Conflict of Interest

The authors declare that the research was conducted in the absence of any commercial or financial relationships that could be construed as a potential conflict of interest.

## Abbreviations

HIF-1: Hypoxia-inducible factor-1
PHD: Prolyl hydroxylase domain-containing enzyme
VHL: von Hippel-Lindau protein
FIH-1: Factor-inhibiting HIF-1α
ERFs: Ethylene response factors
ROS: Reactive oxygen species
NO: Nitric oxide
ETC: Electron transport chain
SREBPs: Sterol regulatory element binding proteins
ER: Endoplasmic reticulum
SCAP: SREBP cleavage-activating protein
PMN: Polymorphonuclear leukocyote
TCA: Tricarboxylic acid
PCO: Plant cysteine oxidase
HRE: Hypoxia-responsive ERF
PAL: Phenylalanine ammonia-lyase
PER: Peroxidase
4-CL: 4-coumerate coenzyme A ligase
CAD: Cinnamyl alcohol dehydrogenase
PDC: Pyruvate decarboxylase
ADH: Alcohol dehydrogenase
HCD: Hypersensitive cell death

## References

[ref1] Aguilar E. A. Turner D. W. Sivasithamparam K. (2000). *Fusarium oxysporum* f. sp. *cubense* inoculation and hypoxia alter peroxidase and phenylalanine ammonia lyase activities in nodal roots of banana cultivars (*Musa* sp.) differing in their susceptibility to *Fusarium* wilt. Aust. J. Bot. 48, 589–596. 10.1071/BT99009

[ref2] Atwell B. Heritage A. (1994). Reduced susceptibility of roots of safflower to *Phytophthora cryptogea* after prior adaptation of roots to hypoxic conditions. Aust. J. Bot. 42, 29–36. 10.1071/BT9940029

[ref3] Babujee L. Apodaca J. Balakrishnan V. Liss P. Kiley P. J. Charkowski A. O.. (2012). Evolution of the metabolic and regulatory networks associated with oxygen availability in two phytopathogenic enterobacteria. BMC Genomics 13:110. 10.1186/1471-2164-13-110, PMID: 22439737 PMC3349551

[ref4] Bailey-Serres J. Chang R. (2005). Sensing and signalling in response to oxygen deprivation in plants and other organisms. Ann. Bot. 96, 507–518. 10.1093/aob/mci206, PMID: 16051633 PMC4247021

[ref5] Barker B. M. Kroll K. Vodisch M. Mazurie A. Kniemeyer O. Cramer R. A. (2012). Transcriptomic and proteomic analyses of the *Aspergillus fumigatus* hypoxia response using an oxygen-controlled fermenter. BMC Genomics 13:62. 10.1186/1471-2164-13-62, PMID: 22309491 PMC3293747

[ref6] Bedogni B. Powell M. B. (2009). Hypoxia, melanocytes and melanoma – survival and tumor development in the permissive microenvironment of the skin. Pigment Cell Melanoma Res. 22, 166–174. 10.1111/j.1755-148X.2009.00553.x, PMID: 19222803 PMC3038250

[ref7] Bien C. M. Espenshade P. J. (2010). Sterol regulatory element binding proteins in fungi: hypoxic transcription factors linked to pathogenesis. Eukaryot. Cell 9, 352–359. 10.1128/EC.00358-09, PMID: 20118213 PMC2837984

[ref8] Bischof M. Eichmann R. Hückelhoven R. (2011). Pathogenesis-associated transcriptional patterns in Triticeae. J. Plant Physiol. 168, 9–19. 10.1016/j.jplph.2010.06.013, PMID: 20674077

[ref9] Blatzer M. Barker B. M. Willger S. D. Beckmann N. Blosser S. J. Cornish E. J.. (2011). SREBP coordinates iron and ergosterol homeostasis to mediate triazole drug and hypoxia responses in the human fungal pathogen *Aspergillus fumigatus*. PLoS Genet. 7:e1002374. 10.1371/journal.pgen.1002374, PMID: 22144905 PMC3228822

[ref10] Burgess T. McComb J. A. Colquhoun I. Hardy G. E. S. (1999). Increased susceptibility of *Eucalyptus marginata* to stem infection by *Phytophthora cinnamomi* resulting from root hypoxia. Plant Pathol. 48, 797–806. 10.1046/j.1365-3059.1999.00396.x

[ref11] Burr R. Espenshade P. J. (2017). Oxygen-responsive transcriptional regulation of lipid homeostasis in fungi: implications for anti-fungal drug development. Semin. Cell Dev. Biol. 81, 110–120. 10.1016/j.semcdb.2017.08.043 28851600 PMC5826825

[ref12] Butler G. (2013). Hypoxia and gene expression in eukaryotic microbes. Annu. Rev. Microbiol. 67, 291–312. 10.1146/annurev-micro-092412-155658, PMID: 23808338

[ref13] Camy C. Dreyer E. Delatour C. Marçais B. (2003). Responses of the root rot fungus *Collybia fusipes* to soil waterlogging and oxygen availability. Mycol. Res. 107, 1103–1109. 10.1017/S095375620300830X, PMID: 14563138

[ref14] Castello P. R. David P. S. McClure T. Crook Z. Poyton R. O. (2006). Mitochondrial cytochrome oxidase produces nitric oxide under hypoxic conditions: implications for oxygen sensing and hypoxic signaling in eukaryotes. Cell Metab. 3, 277–287. 10.1016/j.cmet.2006.02.011, PMID: 16581005

[ref15] Chang Y. C. Bien C. M. Lee H. Espenshade P. J. Kwon-Chung K. J. (2007). Sre1p, a regulator of oxygen sensing and sterol homeostasis, is required for virulence in *Cryptococcus neoformans*. Mol. Microbiol. 64, 614–629. 10.1111/j.1365-2958.2007.05676.x, PMID: 17462012

[ref16] Choi J. Chung H. Lee G. -W. Koh S. -K. Chae S. -K. Lee Y. -H. (2015). Genome-wide analysis of hypoxia-responsive genes in the rice blast fungus, *Magnaporthe oryzae*. PLoS One 10:e0134939. 10.1371/journal.pone.0134939, PMID: 26241858 PMC4524601

[ref17] Chun C. D. Liu O. W. Madhani H. D. (2007). A link between virulence and homeostatic responses to hypoxia during infection by the human fungal pathogen *Cryptococcus neoformans*. PLoS Pathog. 3:e22. 10.1371/journal.ppat.0030022, PMID: 17319742 PMC1803011

[ref18] Chung D. Barker B. M. Carey C. C. Merriman B. Werner E. R. Lechner B. E.. (2014). ChIP-seq and in vivo transcriptome analyses of the *Aspergillus fumigatus* SREBP SrbA reveals a new regulator of the fungal hypoxia response and virulence. PLoS Pathog. 10:e1004487. 10.1371/journal.ppat.1004487, PMID: 25375670 PMC4223079

[ref19] Chung D. Haas H. Cramer R. A. (2012). Coordination of hypoxia adaptation and iron homeostasis in human pathogenic fungi. Front. Microbiol. 3:381. 10.3389/fmicb.2012.00381, PMID: 23133438 PMC3490150

[ref20] Chung H. Kim S. Kim K. -T. Hwang B. -G. Kim H. -J. Lee S. -J.. (2019). A novel approach to investigate hypoxic microenvironment during rice colonization by *Magnaporthe oryzae*. Environ. Microbiol. 21, 1151–1169. 10.1111/1462-2920.14563, PMID: 30773773

[ref21] Cummins E. P. Taylor C. T. (2005). Hypoxia-responsive transcription factors. Pflugers Arch. 450, 363–371. 10.1007/s00424-005-1413-7, PMID: 16007431

[ref22] Davison E. Tay F. (1987). The effect of waterlogging on infection of *Eucalyptus marginata* seedlings by *Phytophthora cinnamomi*. New Phytol. 105, 585–594. 10.1111/j.1469-8137.1987.tb00896.x

[ref23] De Boer S. Kelman A. (1978). Influence of oxygen concentration and storage factors on susceptibility of potato tubers to bacterial soft rot (*Erwinia carotovora*). Potato Res. 21, 65–79. 10.1007/BF02362262

[ref24] Dirmeier R. O’Brien K. M. Engle M. Dodd A. Spears E. Poyton R. O. (2002). Exposure of yeast cells to anoxia induces transient oxidative stress – implications for the induction of hypoxic genes. J. Biol. Chem. 277, 34773–34784. 10.1074/jbc.M203902200, PMID: 12089150

[ref25] DuBois J. C. Pasula R. Dade J. E. Smulian A. G. (2016). Yeast transcriptome and in vivo hypoxia detection reveals *Histoplasma capsulatum* response to low oxygen tension. Med. Mycol. 54, 40–58. 10.1093/mmy/myv073, PMID: 26483436

[ref26] DuBois J. C. Smulian A. G. (2016). Sterol regulatory element binding protein (Srb1) is required for hypoxic adaptation and virulence in the dimorphic fungus *Histoplasma capsulatum*. PLoS One 11:e0163849. 10.1371/journal.pone.0163849, PMID: 27711233 PMC5053422

[ref27] Emerling B. M. Chandel N. S. (2005). Oxygen sensing: getting pumped by sterols. Sci. STKE 2005:pe30. 10.1126/stke.2892005pe30, PMID: 15972699

[ref28] Ernst J. F. Tielker D. (2009). Responses to hypoxia in fungal pathogens. Cell. Microbiol. 11, 183–190. 10.1111/j.1462-5822.2008.01259.x, PMID: 19016786

[ref29] Friedman E. S. Bittinger K. Esipova T. V. Hou L. K. Chau L. L. Jiang J.. (2018). Microbes vs. chemistry in the origin of the anaerobic gut lumen. Proc. Natl. Acad. Sci. U. S. A. 115, 4170–4175. 10.1073/pnas.1718635115, PMID: 29610310 PMC5910840

[ref30] Galea A. M. Brown A. J. (2009). Special relationship between sterols and oxygen: were sterols an adaptation to aerobic life? Free Radic. Biol. Med. 47, 880–889. 10.1016/j.freeradbiomed.2009.06.027, PMID: 19559787

[ref31] Geigenberger P. (2003). Response of plant metabolism to too little oxygen. Curr. Opin. Plant Biol. 6, 247–256. 10.1016/S1369-5266(03)00038-4, PMID: 12753974

[ref32] Geigenberger P. Fernie A. R. Gibon Y. Christ M. Stitt M. (2000). Metabolic activity decreases as an adaptive response to low internal oxygen in growing potato tubers. Biol. Chem. 381, 723–740. 10.1515/BC.2000.093, PMID: 11030430

[ref33] Giaccia A. Siim B. G. Johnson R. S. (2003). HIF-1 as a target for drug development. Nat. Rev. Drug Discov. 2, 803–811. 10.1038/nrd1199, PMID: 14526383

[ref34] Gibbs D. J. Lee S. C. Isa N. M. Gramuglia S. Fukao T. Bassel G. W.. (2011). Homeostatic response to hypoxia is regulated by the N-end rule pathway in plants. Nature 479, 415–418. 10.1038/nature10534, PMID: 22020279 PMC3223408

[ref35] Grahl N. Cramer R. A. Jr. (2010). Regulation of hypoxia adaptation: an overlooked virulence attribute of pathogenic fungi? Med. Mycol. 48, 1–15. 10.3109/13693780902947342, PMID: 19462332 PMC2898717

[ref36] Grahl N. Puttikamonkul S. Macdonald J. M. Gamcsik M. P. Ngo L. Y. Hohl T. M.. (2011). In vivo hypoxia and a fungal alcohol dehydrogenase influence the pathogenesis of invasive pulmonary aspergillosis. PLoS Pathog. 7:e1002145. 10.1371/journal.ppat.1002145, PMID: 21811407 PMC3141044

[ref37] Grahl N. Shepardson K. M. Chung D. Cramer R. A. (2012). Hypoxia and fungal pathogenesis: to air or not to air? Eukaryot. Cell 11, 560–570. 10.1128/EC.00031-12, PMID: 22447924 PMC3346435

[ref38] Gravot A. Richard G. Lime T. Lemarie S. Jubault M. Lariagon C.. (2016). Hypoxia response in *Arabidopsis* roots infected by *Plasmodiophora brassicae* supports the development of clubroot. BMC Plant Biol. 16:251. 10.1186/s12870-016-0941-y, PMID: 27835985 PMC5106811

[ref39] Haque N. Rahman M. T. Abu Kasim N. H. Alabsi A. M. (2013). Hypoxic culture conditions as a solution for mesenchymal stem cell based regenerative therapy. Sci. World J. 2013:632972. 10.1155/2013/632972, PMID: 24068884 PMC3771429

[ref40] Heritage A. Duniway J. (1985). “Influence of depleted oxygen supply on *Phytophthora* root rot of safflower in nutrient solution” in Ecology and management of soil-borne plant pathogens. eds. Parker C. A. Rovira A. D. Moore K. J. Wong P. T. W. Kollmorgen J. F. (St. Paul, MN, USA: American Phytopathological Society), 199–202.

[ref41] Hillmann F. Shekhova E. Kniemeyer O. (2015). Insights into the cellular responses to hypoxia in filamentous fungi. Curr. Genet. 61, 441–455. 10.1007/s00294-015-0487-9, PMID: 25911540

[ref42] Hückelhoven R. Fodor J. Preis C. Kogel K. -H. (1999). Hypersensitive cell death and papilla formation in barley attacked by the powdery mildew fungus are associated with hydrogen peroxide but not with salicylic acid accumulation. Plant Physiol. 119, 1251–1260. 10.1104/pp.119.4.1251, PMID: 10198083 PMC32009

[ref43] Hugouvieux-Cotte-Pattat N. Dominguez H. Robert-Baudouy J. (1992). Environmental conditions affect transcription of the pectinase genes of *Erwinia chrysanthemi* 3937. J. Bacteriol. 174, 7807–7818. 10.1128/JB.174.23.7807-7818.1992, PMID: 1447147 PMC207497

[ref44] Ibrahim-Granet O. Jouvion G. Hohl T. M. Droin-Bergere S. Philippart F. Kim O. Y.. (2010). In vivo bioluminescence imaging and histopathopathologic analysis reveal distinct roles for resident and recruited immune effector cells in defense against invasive aspergillosis. BMC Microbiol. 10:105. 10.1186/1471-2180-10-105, PMID: 20377900 PMC2859869

[ref45] Ingavale S. S. Chang Y. C. Lee H. McClelland C. M. Leong M. L. Kwon-Chung K. J. (2008). Importance of mitochondria in survival of *Cryptococcus neoformans* under low oxygen conditions and tolerance to cobalt chloride. PLoS Pathog. 4:e1000155. 10.1371/journal.ppat.1000155, PMID: 18802457 PMC2528940

[ref46] Javid M. Zhang P. Taylor P. W. Pethybridge S. J. Groom T. Nicolas M. E. (2013). Interactions between waterlogging and ray blight in pyrethrum. Crop Pasture Sci. 64, 726–735. 10.1071/CP13064

[ref47] Kobayashi M. Matsuo Y. Takimoto A. Suzuki S. Maruo F. Shoun H. (1996). Denitrification, a novel type of respiratory metabolism in fungal mitochondrion. J. Biol. Chem. 271, 16263–16267. 10.1074/jbc.271.27.16263, PMID: 8663075

[ref48] Komatsu H. Cook C. Wang C. H. Medrano L. Lin H. Kandeel F.. (2017). Oxygen environment and islet size are the primary limiting factors of isolated pancreatic islet survival. PLoS One 12:e0183780. 10.1371/journal.pone.0183780, PMID: 28832685 PMC5568442

[ref49] Kowalski C. H. Beattie S. R. Fuller K. K. McGurk E. A. Tang Y. -W. Hohl T. M.. (2016). Heterogeneity among isolates reveals that fitness in low oxygen correlates with *Aspergillus fumigatus* virulence. MBio 7:e01515-16. 10.1128/mBio.01515-16, PMID: 27651366 PMC5040115

[ref50] Kowalski C. H. Kerkaert J. D. Liu K. -W. Bond M. C. Hartmann R. Nadell C. D.. (2019). Fungal biofilm morphology impacts hypoxia fitness and disease progression. Nat. Microbiol. 4, 2430–2441. 10.1038/s41564-019-0558-7, PMID: 31548684 PMC7396965

[ref51] Kroll K. Pähtz V. Hillmann F. Vaknin Y. Schmidt-Heck W. Roth M.. (2014). Identification of hypoxia-inducible target genes of *Aspergillus fumigatus* by transcriptome analysis reveals cellular respiration as an important contributor to hypoxic survival. Eukaryot. Cell 13, 1241–1253. 10.1128/EC.00084-14, PMID: 25084861 PMC4187615

[ref52] Kuan T. Erwin D. (1980). Predisposition effect of water saturation of soil on *Phytophthora* root rot of alfalfa. Phytopathology 70, 981–986. 10.1094/Phyto-70-981

[ref53] Licausi F. Kosmacz M. Weits D. A. Giuntoli B. Giorgi F. M. Voesenek L. A. C. J.. (2011). Oxygen sensing in plants is mediated by an N-end rule pathway for protein destabilization. Nature 479, 419–422. 10.1038/nature10536, PMID: 22020282

[ref54] Lima P. D. S. Chung D. Bailão A. M. Cramer R. A. Soares C. M. D. A. (2015). Characterization of the *Paracoccidioides* hypoxia response reveals new insights into pathogenesis mechanisms of this important human pathogenic fungus. PLoS Negl. Trop. Dis. 9:e0004282. 10.1371/journal.pntd.0004282, PMID: 26659387 PMC4686304

[ref55] Lisicka W. Fikowicz-Krosko J. Jafra S. Narajczyk M. Czaplewska P. Czajkowski R. (2018). Oxygen availability influences expression of *Dickeya solani* genes associated with virulence in potato (*Solanum tuberosum* L.) and chicory (*Cichorium intybus* L.). Front. Plant Sci. 9:374. 10.3389/fpls.2018.00374, PMID: 29619040 PMC5872005

[ref56] Lopes J. P. Stylianou M. Backman E. Holmberg S. Jass J. Claesson R.. (2018). Evasion of immune surveillance in low oxygen environments enhances *Candida albicans* virulence. MBio 9:e02120-18. 10.1128/mBio.02120-18, PMID: 30401781 PMC6222133

[ref57] Losada L. Barker B. M. Pakala S. Pakala S. Joardar V. Zafar N.. (2014). Large-scale transcriptional response to hypoxia in *Aspergillus fumigatus* observed using RNAseq identifies a novel hypoxia regulated ncRNA. Mycopathologia 178, 331–339. 10.1007/s11046-014-9779-8, PMID: 24996522 PMC4239182

[ref58] MacPherson S. Akache B. Weber S. De Deken X. Raymond M. Turcotte B. (2005). *Candida albicans* zinc cluster protein Upc2p confers resistance to antifungal drugs and is an activator of ergosterol biosynthetic genes. Antimicrob. Agents Chemother. 49, 1745–1752. 10.1128/AAC.49.5.1745-1752.2005, PMID: 15855491 PMC1087678

[ref59] Majmundar A. J. Wong W. H. J. Simon M. C. (2010). Hypoxia-inducible factors and the response to hypoxic stress. Mol. Cell 40, 294–309. 10.1016/j.molcel.2010.09.022, PMID: 20965423 PMC3143508

[ref60] McDonald K. L. Sutherland M. W. Guest D. I. (2002). Temporary hypoxia suppresses the oxidative burst and subsequent hypersensitive cell death in cells of tobacco and soybean challenged with zoospores of incompatible isolates of *Phytophthora* species. Physiol. Mol. Plant Pathol. 61, 133–140. 10.1006/pmpp.2002.0423

[ref61] Moslemi A. Ades P. K. Groom T. Nicolas M. E. Taylor P. W. (2018). Influence of waterlogging on growth of pyrethrum plants infected by the crown and root rot pathogens, *Fusarium oxysporum*, *Fusarium avenaceum* and *Paraphoma vinacea*. Australas. Plant Pathol. 47, 205–213. 10.1007/s13313-018-0547-y

[ref62] Mulhern S. M. Logue M. E. Butler G. (2006). *Candida albicans* transcription factor Ace2 regulates metabolism and is required for filamentation in hypoxic conditions. Eukaryot. Cell 5, 2001–2013. 10.1128/EC.00155-06, PMID: 16998073 PMC1694816

[ref63] Mustroph A. Lee S. C. Oosumi T. Zanetti M. E. Yang H. Ma K.. (2010). Cross-kingdom comparison of transcriptomic adjustments to low-oxygen stress highlights conserved and plant-specific responses. Plant Physiol. 152, 1484–1500. 10.1104/pp.109.151845, PMID: 20097791 PMC2832244

[ref64] Narsai R. Rocha M. Geigenberger P. Whelan J. van Dongen J. T. (2011). Comparative analysis between plant species of transcriptional and metabolic responses to hypoxia. New Phytol. 190, 472–487. 10.1111/j.1469-8137.2010.03589.x, PMID: 21244431

[ref65] Niehoff K. Barnikol W. (1999). “A new measuring device for non-invasive determination of oxygen partial pressure and oxygen conductance of the skin and other tissues” in Oxygen transport to tissue XXI. Advances in experimental medicine and biology. Vol. 471 eds. Eke A. Delpy D. T. (Boston, MA: Springer), 705–714. 10.1007/978-1-4615-4717-4_81 10659205

[ref66] Nizet V. Johnson R. S. (2009). Interdependence of hypoxic and innate immune responses. Nat. Rev. Immunol. 9, 609–617. 10.1038/nri2607, PMID: 19704417 PMC4343208

[ref67] Ober E. S. Sharp R. E. (1996). A microsensor for direct measurement of O_2_ partial pressure within plant tissues. J. Exp. Bot. 47, 447–454. 10.1093/jxb/47.3.447

[ref68] Osborne T. F. (2011). Sterols for oxygen: the metabolic burden of microbial SREBP. Mol. Cell 44, 172–174. 10.1016/j.molcel.2011.10.004, PMID: 22017866 PMC3226767

[ref69] Paster N. Barkai-golan R. Calderon M. (1986). Control of T-2 toxin production using atmospheric gases. J. Food Prot. 49, 615–617. 10.4315/0362-028X-49.8.615, PMID: 30959694

[ref70] Paster N. Lisker N. (1985). “Effects of controlled atmospheres on *Penicillium patulum* growth and patulin production” in Trichothecenes and other mycotoxins. ed. Lacy J. (New York: John Wiley & Sons).

[ref71] Poyton R. O. Castello P. R. Ball K. A. Woo D. K. Pan N. (2009). Mitochondria and hypoxic signaling a new view. Ann. N. Y. Acad. Sci. 1177, 48–56. 10.1111/j.1749-6632.2009.05046.x, PMID: 19845606

[ref72] Pradhan A. Avelar G. M. Bain J. M. Childers D. S. Larcombe D. E. Netea M. G.. (2018). Hypoxia promotes immune evasion by triggering β-glucan masking on the *Candida albicans* cell surface via mitochondrial and cAMP-protein kinase A signaling. MBio 9:e01318-18. 10.1128/mBio.01318-18, PMID: 30401773 PMC6222127

[ref73] Proels R. K. Westermeier W. Hückelhoven R. (2011). Infection of barley with the parasitic fungus *Blumeria graminis* f. sp. *hordei* results in the induction of *HvADH1* and *HvADH2*. Plant Signal. Behav. 6, 1584–1587. 10.4161/psb.6.10.16889, PMID: 21918380 PMC3256390

[ref74] Pucciariello C. Perata P. (2017). New insights into reactive oxygen species and nitric oxide signalling under low oxygen in plants. Plant Cell Environ. 40, 473–482. 10.1111/pce.12715, PMID: 26799776

[ref75] Rodeghiero M. Rubol S. Bellin A. Turco E. Molinatto G. Gianelle D.. (2018). High resolution assessment of spatio-temporal changes in O_2_ concentration in root-pathogen interaction. Front. Microbiol. 9:1491. 10.3389/fmicb.2018.01491, PMID: 30026738 PMC6041416

[ref76] Rolletschek H. Borisjuk L. Koschorreck M. Wobus U. Weber H. (2002). Legume embryos develop in a hypoxic environment. J. Exp. Bot. 53, 1099–1107. 10.1093/jexbot/53.371.1099, PMID: 11971921

[ref77] Rumeau D. Maher E. A. Kelman A. Showalter A. M. (1990). Extensin and phenylalanine ammonia-lyase gene-expression altered in potato-tubers in response to wounding, hypoxia, and *Erwinia carotovora* infection. Plant Physiol. 93, 1134–1139. 10.1104/pp.93.3.1134, PMID: 16667569 PMC1062642

[ref78] Sasidharan R. Mustroph A. (2011). Plant oxygen sensing is mediated by the N-end rule pathway: a milestone in plant anaerobiosis. Plant Cell 23, 4173–4183. 10.1105/tpc.111.093880, PMID: 22207573 PMC3269858

[ref79] Scholes J. Lee P. Horton P. Lewis D. (1994). Invertase: understanding changes in the photosynthetic and carbohydrate metabolism of barley leaves infected with powdery mildew. New Phytol. 126, 213–222. 10.1111/j.1469-8137.1994.tb03939.x

[ref80] Semenza G. L. (1998). Hypoxia-inducible factor 1: master regulator of O_2_ homeostasis. Curr. Opin. Genet. Dev. 8, 588–594. 10.1016/S0959-437X(98)80016-6, PMID: 9794818

[ref81] Setiadi E. R. Doedt T. Cottier F. Noffz C. Ernst J. F. (2006). Transcriptional response *Candida albicans* to hypoxia: linkage of oxygen sensing and Efg1p-regulatory networks. J. Mol. Biol. 361, 399–411. 10.1016/j.jmb.2006.06.040, PMID: 16854431

[ref82] Shivas R. G. Wood P. M. Darcey M. W. Pegg K. G. (1995). First record of *Fusarium oxysporum* f. sp. *cubense* on cavendish bananas in Western Australia. Australas. Plant Pathol. 24, 38–43. 10.1071/APP9950038

[ref83] Silver P. M. Oliver B. G. White T. C. (2004). Role of *Candida albicans* transcription factor Upc2p in drug resistance and sterol metabolism. Eukaryot. Cell 3, 1391–1397. 10.1128/EC.3.6.1391-1397.2004, PMID: 15590814 PMC539032

[ref84] Stichternoth C. Ernst J. F. (2009). Hypoxic adaptation by Efg1 regulates biofilm formation by *Candida albicans*. Appl. Environ. Microbiol. 75, 3663–3672. 10.1128/AEM.00098-09, PMID: 19346360 PMC2687269

[ref85] Swarbrick P. J. Schulze-lefert P. Scholes J. D. (2006). Metabolic consequences of susceptibility and resistance (race-specific and broad-spectrum) in barley leaves challenged with powdery mildew. Plant Cell Environ. 29, 1061–1076. 10.1111/j.1365-3040.2005.01472.x, PMID: 17080933

[ref86] Synnott J. M. Guida A. Mulhern-Haughey S. Higgins D. G. Butler G. (2010). Regulation of the hypoxic response in *Candida albicans*. Eukaryot. Cell 9, 1734–1746. 10.1128/EC.00159-10, PMID: 20870877 PMC2976306

[ref87] Takaya N. (2009). Response to hypoxia, reduction of electron acceptors, and subsequent survival by filamentous fungi. Biosci. Biotechnol. Biochem. 73, 1–8. 10.1271/bbb.80487, PMID: 19129650

[ref88] Tang G. Zhang C. Ju Z. Zheng S. Wen Z. Xu S.. (2018). The mitochondrial membrane protein FgLetm1 regulates mitochondrial integrity, production of endogenous reactive oxygen species and mycotoxin biosynthesis in *Fusarium graminearum*. Mol. Plant Pathol. 19, 1595–1611. 10.1111/mpp.12633, PMID: 29077257 PMC6637989

[ref89] Valeri M. C. Novi G. Weits D. A. Mensuali A. Perata P. Loreti E. (2020). *Botrytis cinerea* induces local hypoxia in *Arabidopsis* leaves. New Phytol. 10.1111/nph.16513, PMID: [Epub ahead of print] 32124454 PMC7754360

[ref90] van Dongen J. T. Schurr U. Pfister M. Geigenberger P. (2003). Phloem metabolism and function have to cope with low internal oxygen. Plant Physiol. 131, 1529–1543. 10.1104/pp.102.017202, PMID: 12692313 PMC166912

[ref91] Waypa G. B. Smith K. A. Schumacker P. T. (2016). O_2_ sensing, mitochondria and ROS signaling: the fog is lifting. Mol. Asp. Med. 47-48, 76–89. 10.1016/j.mam.2016.01.002, PMID: 26776678 PMC4750107

[ref92] Weits D. A. Giuntoli B. Kosmacz M. Parlanti S. Hubberten H. -M. Riegler H.. (2014). Plant cysteine oxidases control the oxygen-dependent branch of the N-end-rule pathway. Nat. Commun. 5:3425. 10.1038/ncomms4425, PMID: 24599061 PMC3959200

[ref93] Wilcox W. (1993). Incidence and severity of crown and root rots on four apple rootstocks following exposure to *Phytophthora* species and waterlogging. J. Am. Soc. Hortic. Sci. 118, 63–67. 10.21273/JASHS.118.1.63

[ref94] Wilcox W. F. Mircetich S. M. (1985). Effects of flooding duration on the development of *Phytophthora* root and crown rots of cherry. Phytopathology 75, 1451–1455. 10.1094/Phyto-75-1451

[ref95] Willger S. D. Puttikamonkul S. Kim K. H. Burritt J. B. Grahl N. Metzler L. J.. (2008). A sterol-regulatory element binding protein is required for cell polarity, hypoxia adaptation, azole drug resistance, and virulence in *Aspergillus fumigatus*. PLoS Pathog. 4:e1000200. 10.1371/journal.ppat.1000200, PMID: 18989462 PMC2572145

[ref96] Xu W. Peng J. Li D. Tsui C. K. Long Z. Wang Q.. (2018). Transcriptional profile of the human skin pathogenic fungus *Mucor irregularis* in response to low oxygen. Med. Mycol. 56, 631–644. 10.1093/mmy/myx081, PMID: 29420826

[ref97] Zabalza A. van Dongen J. T. Froehlich A. Oliver S. N. Faix B. Gupta K. J.. (2009). Regulation of respiration and fermentation to control the plant internal oxygen concentration. Plant Physiol. 149, 1087–1098. 10.1104/pp.108.129288, PMID: 19098094 PMC2633817

[ref98] Zeitouni N. E. Chotikatum S. von Kockritz-Blickwede M. Naim H. Y. (2016). The impact of hypoxia on intestinal epithelial cell functions: consequences for invasion by bacterial pathogens. Mol. Cell. Pediatr. 3:14. 10.1186/s40348-016-0041-y, PMID: 27002817 PMC4803720

